# Efficient COI barcoding using high throughput single-end 400 bp sequencing

**DOI:** 10.1186/s12864-020-07255-w

**Published:** 2020-12-04

**Authors:** Chentao Yang, Yuxuan Zheng, Shangjin Tan, Guanliang Meng, Wei Rao, Caiqing Yang, David G. Bourne, Paul A. O’Brien, Junqiang Xu, Sha Liao, Ao Chen, Xiaowei Chen, Xinrui Jia, Ai-bing Zhang, Shanlin Liu

**Affiliations:** 1 BGI-Shenzhen, Shenzhen, 518083 China; 2 College of Life Sciences, Capital Normal University, Beijing, 100048 China; 3 College of Science and Engineering, James Cook University, Townsville, QLD Australia; 4 Australian Institute of Marine Science, Townsville, QLD Australia; 5 AIMS@JCU, Townsville, QLD Australia; 6 Beijing Advanced Innovation Center for Food Nutrition and Human Health, College of Plant Protection, China Agricultural University, Beijing, 100193 China

**Keywords:** DNA barcode, High-throughput sequencing, MGISEQ-2000, SE400, *COI*, Biodiversity

## Abstract

**Background:**

Over the last decade, the rapid development of high-throughput sequencing platforms has accelerated species description and assisted morphological classification through DNA barcoding. However, the current high-throughput DNA barcoding methods cannot obtain full-length barcode sequences due to read length limitations (e.g. a maximum read length of 300 bp for the Illumina’s MiSeq system), or are hindered by a relatively high cost or low sequencing output (e.g. a maximum number of eight million reads per cell for the PacBio’s SEQUEL II system).

**Results:**

Pooled cytochrome c oxidase subunit I (*COI*) barcodes from individual specimens were sequenced on the MGISEQ-2000 platform using the single-end 400 bp (SE400) module. We present a bioinformatic pipeline, HIFI-SE, that takes reads generated from the 5′ and 3′ ends of the *COI* barcode region and assembles them into full-length barcodes. HIFI-SE is written in Python and includes four function modules of *filter*, *assign*, *assembly* and *taxonomy*. We applied the HIFI-SE to a set of 845 samples (30 marine invertebrates, 815 insects) and delivered a total of 747 fully assembled *COI* barcodes as well as 70 *Wolbachia* and fungi symbionts. Compared to their corresponding Sanger sequences (72 sequences available), nearly all samples (71/72) were correctly and accurately assembled, including 46 samples that had a similarity score of 100% and 25 of ca. 99%.

**Conclusions:**

The HIFI-SE pipeline represents an efficient way to produce standard full-length barcodes, while the reasonable cost and high sensitivity of our method can contribute considerably more DNA barcodes under the same budget. Our method thereby advances DNA-based species identification from diverse ecosystems and increases the number of relevant applications.

## Background

Since it was first proposed by Hebert et al. [1], DNA barcoding has attracted global synergistic efforts resulting in well-curated and centralized reference databases. The Barcode of Life Data systems (BOLD) [2], for example, has been growing into a repository of greater than 11 M barcodes representing 314 K species (accessed in Jun. 2020). The applications of DNA barcoding are wide-ranging and may be used to identify species across different life stages and from various environments (e.g. predator feces [3, 4] and from stomach contents [5]). This, along with the ease of barcoding accessibility and analysis, has led to its use in a wide spectrum of scientific and commercial areas, such as cryptic species discovery [6], biodiversity monitoring [7–9], conservation biology [10], inspection of illegal trade of endangered species [11] and discovery of illegal ingredients in medicine [12].

Barcode sequences have been accumulating rapidly in the last decade, prompting a need to improve the available reference databases as they are currently limited by poor and biased spatial coverage and skewed taxonomic coverage [13–16]. Biodiversity initiatives are often limited by insufficient funding, which makes it difficult to include both morphological identification and DNA-based taxonomic work. Therefore, scientists have been attempting to generate cost-efficient barcode sequences via high-throughput sequencing (HTS) platforms. Reduced costs would increase the accessibility of large-scale genomic studies to researchers, allowing for genome resequencing of hundreds of individuals and in turn improving the identification and taxonomy of wild species, particularly those that are difficult to sample. Furthermore, tissues sampled by minimal or non-invasive methods cannot be identified morphologically and an efficient method for species identification will benefit the sample pre-treatment and selection for large-scale genome resequencing studies.

**Fig. 1:**
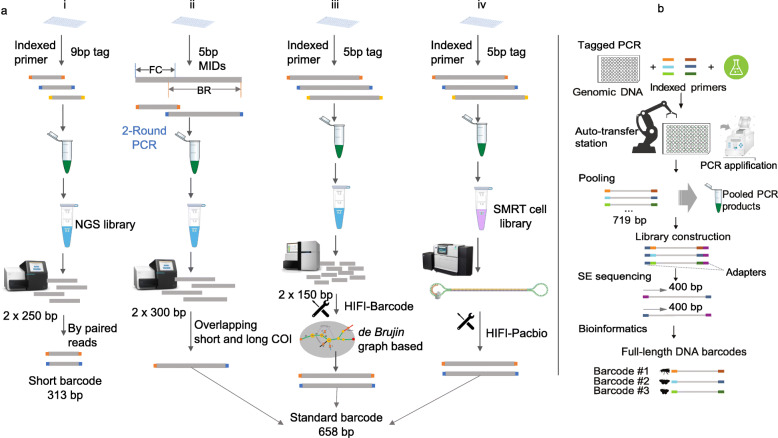
Comparison of different strategies to access *COI* barcode using HTS platforms. The different experimental designs and adopted sequencing strategies fit for sequencing length capacity (**a**). For four main methods of previous studies, (i) and (ii) refer to (Meier, Wong, Srivathsan, & Foo, 2016), (Shokralla et al., 2015), respectively, while (iii) and (iv) refers to (Liu, Yang, Zhou, & Zhou, 2017). The HIFI-SE pipeline can easily and directly obtain the standard *COI* barcode by overlapping single-end 400 bp (**b**)

Current HTS based methods for DNA barcoding are not only cost prohibitive, but are also limited in read length or require extra laboratory workloads. For example, a maximum read length of 300 bp is available on Illumina’s MiSeq platform and only delivers a fraction of the standard barcode [17], while multiple rounds of PCRs [18, 19] or an extra K-mer based assembly step (SOAPBarcode [20]) increases laboratory work and leads to accuracy uncertainty [21] (Fig. 1a). Although long reads from the Single Molecular Real Time (SMRT) sequencing platform or nanopore platform can achieve reliable standard barcode sequences, these are at a higher cost than those HTS based methods [21, 22]. Since a standard DNA barcode (e.g. *COI*) with flanking primers and tags can reach ca. 700 bp in length, the HTS platform offers significant advantages provided it can generate reads of ≥400 bp in length, thus forming a minimum overlap of ~ 80 bp (Fig. 1b), which will allow for accurate *COI* barcode assembly by means of simply connecting the 5′ and 3′ reads.

The MGISEQ platform utilizes a technology called DNBSEQ (https://en.mgitech.cn/products/), which amplifies small fragments of genomic DNA into DNA nanoballs by rolling circle amplification, and determines the DNA nanoballs’ sequence using a refined combinatorial Probe Anchor Synthesis (cPAS) sequencing technology [23]. It generates sequences in FASTQ format with quality scores based on a Phred-33 standard (equivalent to Illumina’s NovaSeq system). Several studies have validated its sequencing quality by comparing its performance with that of Illumina generated sequence data from ancient DNA [24], whole-genome [25] and metagenome sample types [26]. The MGISEQ platform has launched a new sequencing kit capable of single-end 400 bp sequencing - SE400 [27], which offers a simple and reliable way to achieve DNA barcodes efficiently. In this study, we explore the potential of the MGISEQ SE400 sequencing in DNA barcode reference construction and quick species identification, and provide an updated HIFI-SE barcode software package that can generate *COI* barcode assemblies using HTS reads of 400 bp length.

## Results

**Fig. 2:**
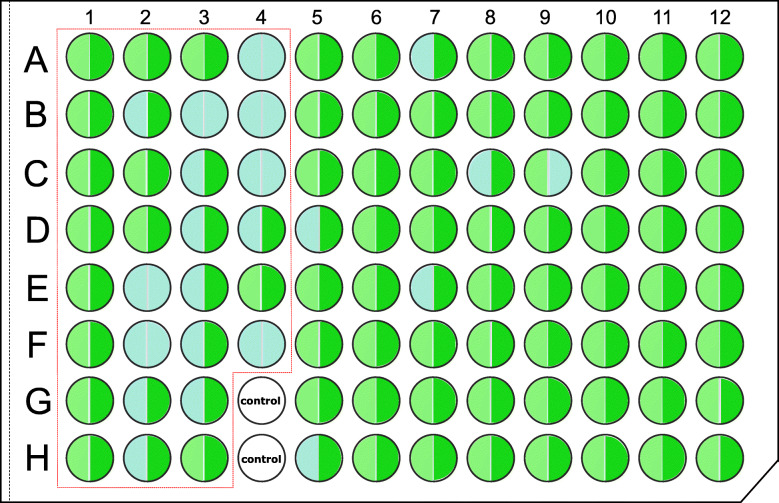
Results of Sanger sequencing (left semicircle) and HIFI-SE barcode assemblies (right semicircle) arranged in a 96-well plate in Plate #1. Gray represents failure; light and dark green represent success of Sanger and HIFI-SE respectively. Marine invertebrate samples are arranged in wells from A01 to F04 (framed by the red tetragon). Insects are arranged in wells from A05 to H12

A total of 73 out of 96 (78%, excluding 2 blanks) samples were successfully sequenced and assembled using Sanger sequencing, with the 21 failed samples referred to as “Barcode failed” samples. Comparatively, for the same 96 samples our pipeline produced a total of 12,745,067 HTS SE400 reads that were retained after quality control and around 77.9% (9,870,823) of reads were assigned to their corresponding samples at either the 5′ or 3′ end. The number of sequences of each sample varied markedly, ranging from 303 to 585,609, with Sanger “barcode failed” samples possessing a lower but insignificant number of reads (Additional file 1: Figure S1). Overall, 86 barcode sequences including 63 insect samples and 23 marine invertebrate samples were achieved using the HIFI-SE pipeline, with 14 out of the 21 Sanger “barcode failed” samples being successfully recovered, leading to an overall success rate of 91.5% (Fig. 2). Conversely, one sample that had a Sanger reference did not successfully assemble using our HIFI-SE pipeline. For the remaining samples, an average of 2,457,295 reads per plate were generated and the output profile and successful assignment ratio were on par with that of Plate #1, producing a total of 661 full-length *COI* barcodes (Additional file 2: Table S3).

When comparing our HIFI-SE assembled sequences to the Sanger reference sequences (72 sequences available), HIFI-SE assemblies showed a high-similarity score for the vast majority of the samples (71/72), including 46 samples that had a sequence similarity of 100% and 25 of ~ 99% (Additional file 2: Table S4). Only one sample displayed a high dissimilarity score to its corresponding Sanger reference sequence. A further examination discovered that its sequence was identical to that of another sample on the same plate, so could have been contaminated by that sample. Read alignment showed that the sites on HIFI-SE assemblies at which mismatches occurred were supported by high read coverage, confirming the accurate recovery of HIFI-SE assemblies (Additional file 1: Figure S2). In addition, HIFI-SE identified a total of 40 ambiguous sites in the Sanger references to specific nucleotides and revealed the heteroplasmy states in some samples (Additional file 1: Figure S2).

**Fig. 3:**
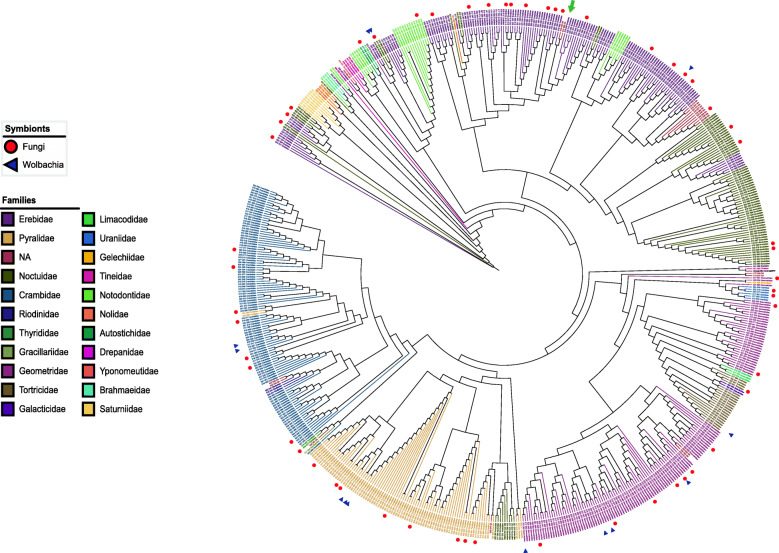
Phylogenetic tree of 660 successful barcodes of moth, with outgroup *Drosophila melanogaster.* The red circle reveals samples containing fungi *COI* barcode, and blue for *Wolbachia COI* barcode. We obtained the taxonomic information of each sample according to that of its best hits on the BOLD database and it may suffer misidentification due to inaccurate records on the database. The phylogeny tree revealed that some specimens could be wrongly identified based on an inadequate database in specific linage. For example, the best hit of #035 in Plate #4 (green arrow) with 100% similarity in BOLD database belongs to *Crambidae* family, however, the second hit with 99.85% similarity belongs to *Erebidae* family. This type of incorrect placement is prone to occur among early-release records, which suggests a new record of specimen need to be carefully reviewed when add to a database, also indicating that morphological identification is still important

For the samples without Sanger references, we first conducted a molecular based taxonomic identification by searching their highly similar records on the BOLD system using the HIFI-SE “*taxonomy*” subprogram. The BOLD database search resulted in a total of 418 samples finding their best hits with similarity scores ≥98% [28–30] and the remaining 243 samples with their best hits with similarity scores ranging from 91.4 to 98% [31, 32]. These sequences represented 21 families of Lepidoptera and an unexpected *Homo sapiens* match (99.86% sequence identity on NCBI), which is likely contamination during wet-lab experiments. To further evaluate the accuracy of the HIFI-SE pipeline, we randomly selected 100 samples which had high-quality photos to identify them morphologically, and then check the conformities between the molecular and morphological identification. For the 91 individuals that successfully produced *COI* barcodes, five records conflicted between the morphological and molecular identification, with the remaining samples being congruent between the two identification approaches (Additional file 1: Figure S3). Since the sequence clusters are supported by many reads, it is possible those taxonomic conflicts resulted from incorrect taxonomic annotations in the BOLD system (Fig. 3 & Additional file 5).

We detected *Wolbachia* derived sequences in 13 samples and fungi derived sequences in 57 samples, including four *Wolbachia* species and 42 fungi species with highly similar records (> 98%) on the BOLD database (Additional file 2: Table S5).

## Discussion

Despite the importance of biodiversity in ecosystem functioning [33], global biodiversity continues to be lost at an unprecedented rate due to climate change and human activities [34]. DNA barcoding has proven effective in accelerating the collection of biodiversity inventories over large geographic and temporal scales, which benefit both researchers and also policy-makers focused on maintaining functioning ecosystems [35]. Burgeoning massive parallel sequencing techniques have driven the cost per nucleotide base down dramatically [36] and facilitated multifaceted approaches to obtain barcode sequences via HTS platforms [20–22]. This has made it possible to generate large amounts of barcode sequences for a tiny fraction of the cost compared to 15 years ago [33, 34, 37].

The HIFI-SE pipeline, that takes advantage of MGISEQ SE400 reads as long as 400 bp, provides an easy, simple and cost-efficient approach to generate barcode sequences from a large number of samples. The 400 bp reads enable an overlap length of ca. 80 bp for most animal *COI* barcode sequences by sequencing both 5’ and 3′ ends. This assembly-by-overlapping step can simplify the barcode assembly process by circumventing the *de Brujin* graph algorithm, which is time-consuming and computationally intensive [38] and can be subject to erroneous pathing when dealing with intricate scenarios.

Currently, high-throughput sequencing platforms (BGI’s MGISEQ/T7 or Illumina’s HiSEQ/NovaSeq) still have advantages in throughput as well as the cost per base/read over the third-generation platforms (PacBio’s SEQUEL II or Oxford Nanopore Technologies’ MinION), and the simplified analysis pipeline based on SE400 sequencing is a further advantage. For example, MGISEQ provides a quote of $650 per lane that can produce ca. 275 million reads compared to a quote of $2000 per cell that can produce < 8 million reads with the PacBio’s latest SEQUEL II release [39]. However, the third-generation platforms have dramatically increased their sequencing throughput in the last 2 years [39] which, together with its advantage of read length, may surpass the next-generation platforms in barcoding related applications using long fragments (e.g. 16S rRNA gene for bacteria). Similarly, ONT’s MinION, a portable and real-time sequencer, can greatly benefit DNA barcoding in terms of speed and flexibility [40]. Thus, while next generation technology is still advantageous for barcoding, third-generation platforms will likely provide useful alternatives in future scenarios.

Two taxonomic groups, marine invertebrates and insects, were sampled in this study to demonstrate the effectiveness of the HIFI-SE approach. The results showed that insects delivered higher barcode recovery ratios (724 out of 815 DNA samples) compared to marine invertebrates (23 out of 30 DNA samples). The relatively lower efficiency of marine invertebrates can be attributed to the biased performance of primer set LCO1490 and HCO2198 [41, 42]. It shows the necessity to improve primer design to cover various phylogenetic lineages in spite of the high sensitivity of HTS methods [43]. The primer’s inadequacy for marine invertebrates was also reflected by excessive short co-amplicons (400 ~ 500 bp) detected in 16 out of 21 Sanger “Barcode failed” samples (Additional file 1: Figure S1), which might be derived from nuclear-encoded mitochondrial DNA (NuMT, [44, 45]) and in turn affect the recovery success of their barcode sequences via both the Sanger sequencing and the HIFI-SE pipeline. Additionally, coral is well known for being difficult in terms of DNA extraction and genomic DNA tends to degrade quite rapidly for many species [46], further contributing to the short co-amplicons. However, this also reveals the strength of our approach by sequencing those samples that are difficult to work with. In addition, we also noticed one assembly (E08 in Additional file 2: Table S4) that showed low similarity to its corresponding Sanger reference was actually cross contamination from another cell (C11 or H12 in Fig. 2). Since we mixed PCR reagents and PCR products using an auto transfer station (Hamilton Microlab® STAR) and sample E08 only contained a read number of 1000, we believe this contamination event could result from pipette failure on the auto transfer station during sample transfer, and a subsequent tag hopping from other samples during library construction and sequencing.

We also noticed that a relative low ratio (69.64%) of clean reads can successfully be assigned to their corresponding samples (Additional file 2: Table S3). A further examination for those unassigned reads found that around 50.8% of them were attributed to chimeras, with primer sequences occurring at unexpected positions on the reads (not at the end), and 49.2% failed to match the tagged primer set due to containing > 2 mismatches. This high proportion of chimeric sequences could be formed during PCR and can be derived by many factors [47], such as PCR ramp and cycles [48, 49], DNA template [50], and DNA polymerases errors [51]. The dual-index method utilized in the current study was shown to be an efficient way to eliminate those problematic PCR artifacts [52]. In addition, we also included an option for a “*taxonomy*” module in HIFI-SE that can BLAST the 5′ and 3′ end of the barcode sequences and then compare taxonomies for consistency to further validate the assembly accuracy. Furthermore, NuMTs can be easily identified by HiFi-SE because most of them are less than 300 bp [53] and thus contain both the forward and reverse primer on a single read. It is also worth noting that two blank samples retrieved *COI* barcodes using the default parameter settings – minimum read number requirement of 10 - reaching a read support number of 13.5 and 12.5, respectively. Thus, the parameter setting for the minimum read number support should be adjusted case by case according to the sequencing depth and the read number of the blank samples.

Although approximately 65% of insect species are estimated to harbor *Wolbachia* [54], we merely detected *Wolbachia* in 13 samples out of 751 moth samples. The low detection ratio could result from the DNA extraction strategy and PCR primer biases, so extra primer sets designed for *Wolbachia* may increase the chances to detect symbiotic bacteria. Further, the fungus detected here were all derived from a single phylum Ascomycota, which contains many well-known fungi that infect and kill insects [55, 56], e.g. *Metarhizium anisopliae*, and fungus in genus *Penicillium*. This taxonomic connection is of interest and deserves further investigation to identify the species interactions which is a focus of major research initiatives such as the BIOSCAN project [37, 57] (https://ibol.org/programs/bioscan/).

## Conclusion

In summary, the HIFI-SE pipeline requires straightforward processing in both sequencing preparation and data analysis, and holds potential to further reduce per unit cost of DNA barcoding while increasing the efficiency and accuracy of the obtained barcodes. Further cost reduction can be achieved by increasing tag length to allow more index combinations, and pooling amplicons using different primer sets. In addition, although we used the *COI* barcode for demonstration, our pipeline is expected to fit other marker genes with a length of 600-750 bp (e.g. V1-V4, V3-V6, and V5-V9 of 16S rRNA gene). Therefore, this new approach can produce standard full-length barcodes cost efficiently, allowing initiatives targeted at DNA barcoding of different biomes to be more achievable, thereby improving our understanding of the biodiversity of global ecosystems or improving DNA based biosecurity programs. Furthermore, the detection of symbiont information using the current protocol provides an efficient way to study the network and adaptive evolution between the hosts and their symbionts or parasites [58–60].

## Methods

### Sample collection and DNA extraction

A total of 845 samples, including marine invertebrates (30 samples) and insects (815 samples) were used to test our COI barcoding pipeline (Additional file 2: Table S1). Marine invertebrates were collected using a hammer and chisel (for sceractinian coral) or sterile razor blades (octocorals and sponges) in May 2017, from Orpheus Island in the central in-shore region of the Great Barrier Reef, under the Marine Parks permit G15/37574.1. Coral tissue was removed from the skeleton using pressurized air from a blow gun into a ziplock bag containing 10 mL of calcium magnesium free artificial seawater (CMFASW; NaCl 26.2 g, KCl 1 g, NaHCO_3_, Milli-Q H_2_O 1 L). Coral tissue blastate was aliquoted into 2 mL microfuge tubes and pelleted in a fixed angle centrifuge at 10,000 x g for 10 min. Pellets were snap frozen and stored at − 80 °C until DNA extraction. All other marine invertebrates were dissected to fit into a 2 mL cryovial, snap frozen and stored at − 80 °C until DNA extraction. Insect samples were collected in August 2017 from the Laohegou Natural Reserve in Sichuan Province and from the Lushan Town, Zhoushan City, Zhejiang Province in China via light trapping. Approximately 0.05 g of coral tissue pellet or marine invertebrate tissue was then used for DNA extraction using the PowerBiofilm DNA Isolation Kit (QIAGEN Pty Ltd., Australia) following the manufacturers protocol. The DNA of insects were extracted using the Glass Fiber Plate method [61], or using the tissue/cell genomic DNA rapid extraction kit (Tiangen Biochemical Technology Co., Ltd., Beijing).

### Tag design, PCR amplification, and sanger sequencing

A total of 96 paired tags were added to both ends of the common *COI* barcode primer set (LCO1490 and HCO2198 [62]) (Additional file 2: Table S2). The tag sequence was 5 bp in length and had ≥2 bp difference from each other. Each PCR reaction (25 μL) contained 1 μL DNA template, 16.2 μL molecular biology grade water, 2.5 μL 10× buffer (Mg^2+^ plus), 2.5 μL dNTP mix (10 mM), 1 μL each forward and reverse primers (10 mM), and 0.3 μL TaKaRa Ex Taq polymerase (5 U/μL) (Takara, Dalian, China). The amplification program included a thermocycling profile of 94 °C for 60s, 5 cycles of 94 °C for 30 s, 45 °C for 40 s, and an extension at 72 °C for 60 s, followed by 35 cycles of 94 °C for 30 s, 51 °C for 40 s, and 72 °C for 60 s, with a final extension at 72 °C for 10 min, and a final on-hold at 12 °C. Amplicons generated from the plate (plate #1) containing both the marine invertebrate and insect species were individually visualized on a 1.2% 96 Agarose E-gel (Biowest Agarose) and Sanger sequenced using an ABI 3730XL sequencer (BGI-Shenzhen) and then assembled using Geneious [63].

### Library construction and sequencing

One microliter of each amplicon was mixed and sent to BGI-Shenzhen for library preparation and sequencing (MGISEQ SE400 module) following the general library construction protocol (Additional file 3), with a minor modification to exclude DNA fragmentation and size selection.

### HIFI-SE: Bioinformatic analysis for SE400 data

**Fig. 4:**
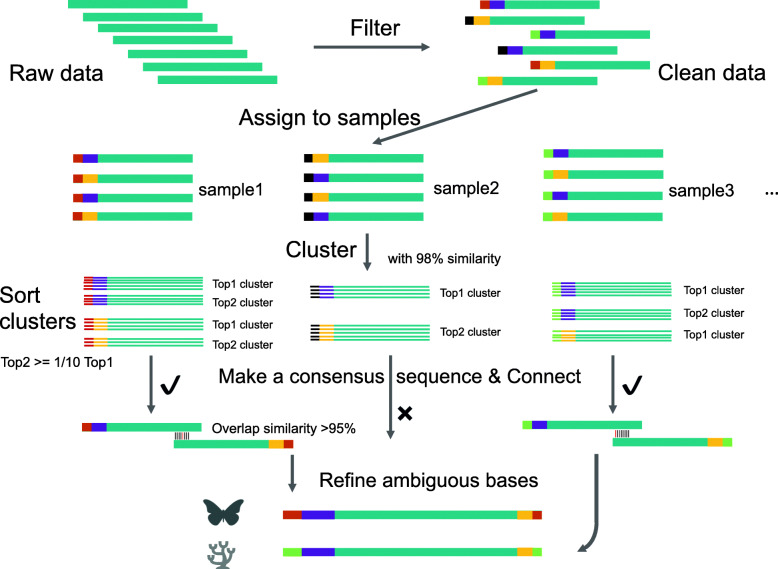
HIFI-SE barcode assembly pipeline. The colored bars from left to right represent tags, primers (purple for 5′ end and orange for 3′ end) and barcode sequences, respectively

To increase accessibility of our newly developed pipeline using the MGISEQ-2000 platform with 400 bp single-end sequencing, we developed a software package, HIFI-SE, which is written in Python and is deposited on PyPI (https://pypi.org/project/HIFI-SE/), consisting of four main function modules of ‘*filter’*, ‘*assign’*, ‘*assembly’* and ‘*taxonomy’* (Fig. 4). Full function instruction and a tutorial are detailed in the software manual (Additional file 4) and briefly outlined below.

#### Data filtering

Removes low quality reads including; 1) reads containing any ambiguous bases (i.e. “N”) and 2) reads with an expected error number *E*^∗^ > 10 with *E*^∗^ calculated using a formula of ${E}^{\ast }=\sum \limits_{i=1}^n{10}^{- Qi/10}$, where n represents sequence length and *Qi* represents base quality (Phred-33 standard) of the *i*^*th*^ base on reads.

#### Read assignment

Reads were demultiplexed by index and classified to the 5′ and 3′ ends according to the primer sequences, allowing one base mismatch in the index region and one base mismatch in the primer region. In addition, since tagged primer sequences are expected to be located at the end of each read, primer sequences found in improper positions (e.g. in the middle) of the reads were regarded as chimeras and removed automatically during the assignment. Finally, all reads were classified into 192 (96*2) groups consisting of both the 5′ and 3′ end for each of the 96 tags.

#### Full-length *COI* barcode assembly

Sequences within each group were first clustered at a 98% similarity using VSEARCH (v2.8.0) [64] and a consensus sequence was built from the most abundant cluster. Additionally, a consensus sequence of the second most abundant cluster was also retained if the number of sequences in that cluster was greater than 1/10 of the top cluster, to identify potential symbionts or parasites. Finally, a minimum sequence number of five for each cluster is needed to guarantee the accuracy of the consensus sequence.

Full-length *COI* barcodes were assembled by connecting the consensus sequences of the 5′ and 3′ ends with an overlap ≥80 bp and a similarity ≥95% (mismatches may exist in the overlapping regions due to reduced read quality when towards the read ends). Mismatches in the overlapped region were determined based on the base frequency calculated from sequences in both ends. The assemblies with correct amino acid translation (without stop codons) and a length of > 650 bp were output as the final results. Users also have the flexibility to run another assembly with an additional parameter in the event samples fail with the default parameter settings, for example, by checking for amino acid translation before clustering (Additional file 4).

#### Taxonomy identification in BOLD

The HIFI-SE pipeline provides an optional step (*taxonomy*) to verify the taxonomic information of the assembled sequences. It can automatically submit assemblies to the BOLD system and retrieve the taxonomic information from the returned searches. Currently, it supports searching of the animal, fungi and plant databases and outputs a user-defined number of BOLD items for each sequence.

### Performance evaluation based on the test samples

#### *COI* barcode retrieval and symbiont detection

We obtained *COI* barcode assemblies for each sample using the HIFI-SE package with default parameter settings. To further detect nontarget *COI* barcodes (e.g. *Wolbachia* and fungi), all the non-targeted clusters with sequence numbers ≥10 were assembled with default settings. We also identified potential symbionts via BLAST searching [65] (version 2.7.1+, E-value ≤1e-5) a manually curated symbiont dataset (COI genes downloaded from NCBI Genbank, https://github.com/comery/HIFI-barcode-SE400/) before submitting all the barcode assemblies to the BOLD system for taxonomic identification.

#### Accuracy estimation

For the samples that were Sanger sequenced, we assembled and achieved the barcode sequences using Geneious [63]. To evaluate the accuracy of HIFI-SE pipeline, the HIFI-SE assemblies were aligned to their Sanger references using MUSCLE (v3.8.31) [66] and then checked for similarities between each. We subsequently aligned the demultiplexed reads to their corresponding HIFI-SE assemblies using BWA (Version: 0.7.17-r1188) [67] to examine read support for sites at which the HIFI-SE assemblies and Sanger sequences were different.

#### Species identification and phylogenetic analysis

Species identification was implemented by HIFI-SE “*taxonomy*” function with a setting of “-n 5 (output five best hits)”. We inferred the phylogenetic relationship for all lepidopteran *COI* barcode sequences using IQ-TREE (version 1.6.5) [68] with *Drosophila melanogaster* used as an outgroup after alignment using MAFFT (v7.245) [69] with the parameters of *“--localpair --maxiterate 16 --phylipout --reorder*”.

## Supplementary Information

**Additional file 1: Figure S1.** Read counts of the Sanger barcode failed samples. Stars indicate samples of which short amplicon(s) was detected in the HIFI assemblies. Short amplicons are those clusters of abundance > 10 and of length < 600 bp. The bar plot demonstrates the number of assigned reads for the barcode failed samples. The red dashed line shows the average value of all the successful samples and no significant difference was detected between the two groups (*P* value of 0.232, Student’s t-Test). **Figure S2.** Discrepancies between Sanger sequences and HIFI-SE barcodes. Entropy weight was calculated based on the strength of read depth by aligning the SE400 reads onto the assembled HIFI-SE barcodes, showing differences between ambiguous Sanger base-calling and specific nucleotide identified in HIFI-SE barcodes (A) and potential heteroplasmy (B). In addition, several N bases were present of insertion in Sanger sequence (C), also two N bases in HIFI sequences (D). **Figure S3.** Comparison of molecular and morphological identification.

**Additional file 2: Table S1.** Sequence of the tagged primers. **Table S2.** Sample Information. **Table S3.** Statistical results of data output and *COI* barcode recovery. **Table S4.** Accuracy results of HIFI-SE barcodes compared with Sanger. **Table S5.** *Wolbachia* and fungi sequences detected from moth samples.

**Additional file 3.** Library construction protocol of MGISEQ-2000 SE400 module.

**Additional file 4.** The manual of HIFI-SE package.

**Additional file 5.** A note for taxonomy identification issue of sample #035 in plate #4.

## Data Availability

The datasets generated and/or analyzed during the current study are available in the CNGB Nucleotide Sequence Archive (CNSA: https://db.cngb.org/cnsa; accession number CNP0000195, and the EMBL repository (PRJEB29212, ERP111495). The HIFI-SE program and symbiont dataset used in this study were deposited on Github (https://github.com/comery/HIFI-barcode-SE400).

## Abbreviations

SE400: Single-end 400 bp long read sequencing
*COI*: Cytochrome *c* oxidase subunit I
BOLD: The Barcode of Life Data systems
HTS: High-throughput sequencing
SMRT: Single Molecular Real Time
HIFI-SE: High-throughput and high-fidelity DNA barcoding analysis pipeline for single-end 400 bp long reads
