# Supplementary figures and images for: Efficient COI barcoding using high throughput single-end 400 bp sequencing
Source: BMC Genomics. 2020 Dec 4;21:862. doi: 10.1186/s12864-020-07255-w (PMC7716423; doi:10.1186/s12864-020-07255-w)

Figure S1

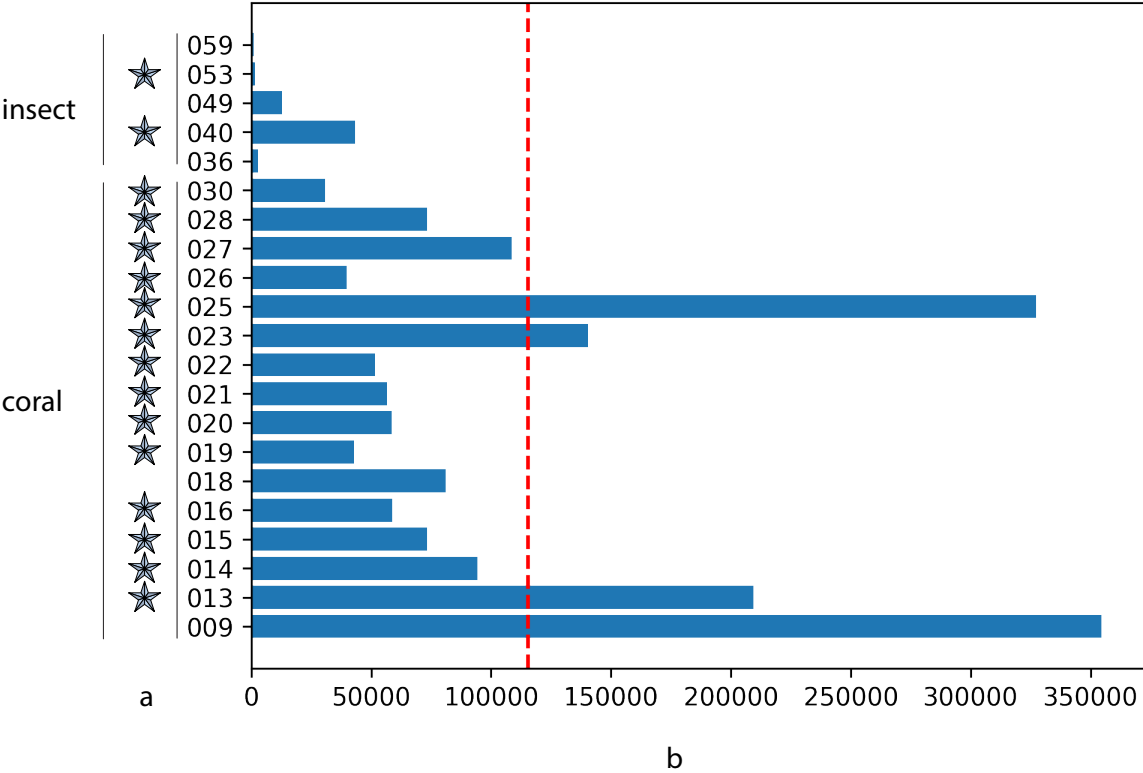

Figure S2

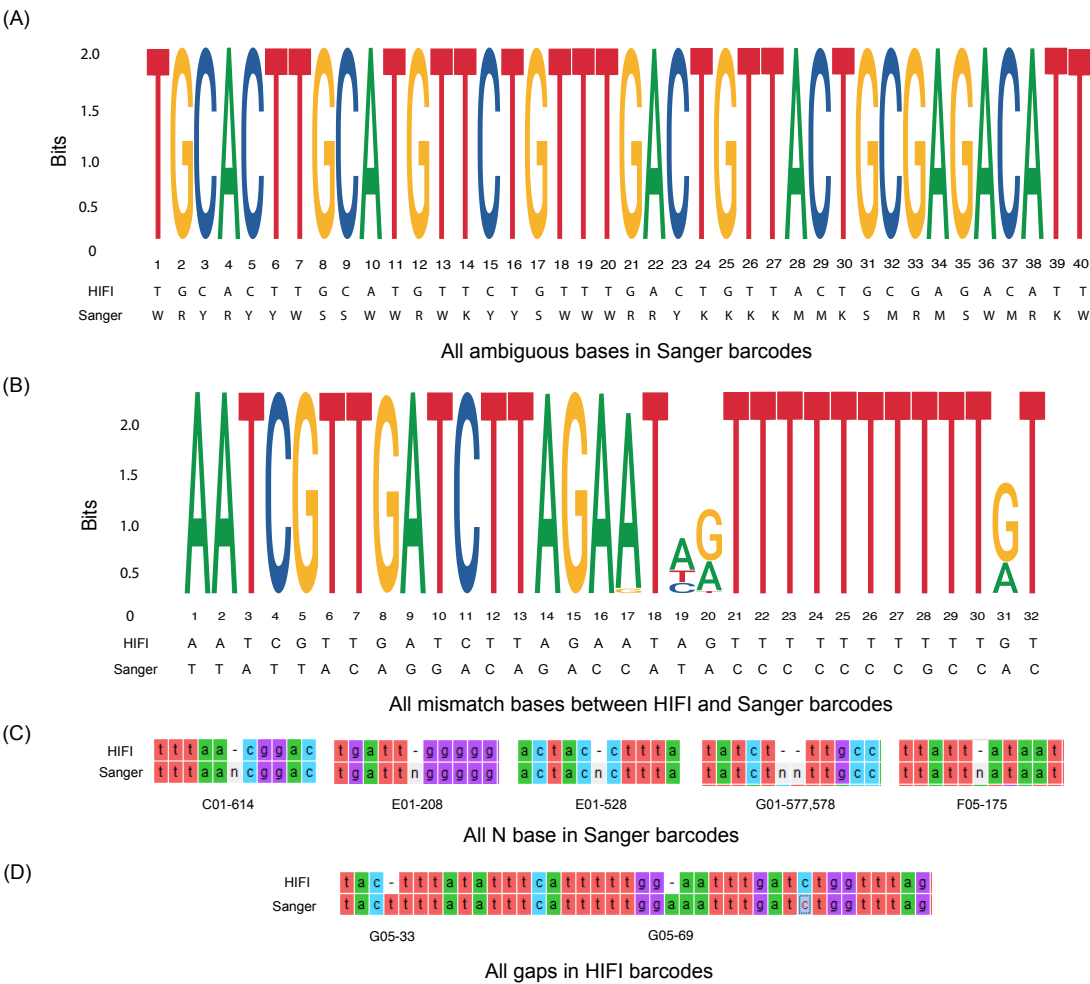

**Figure S3**

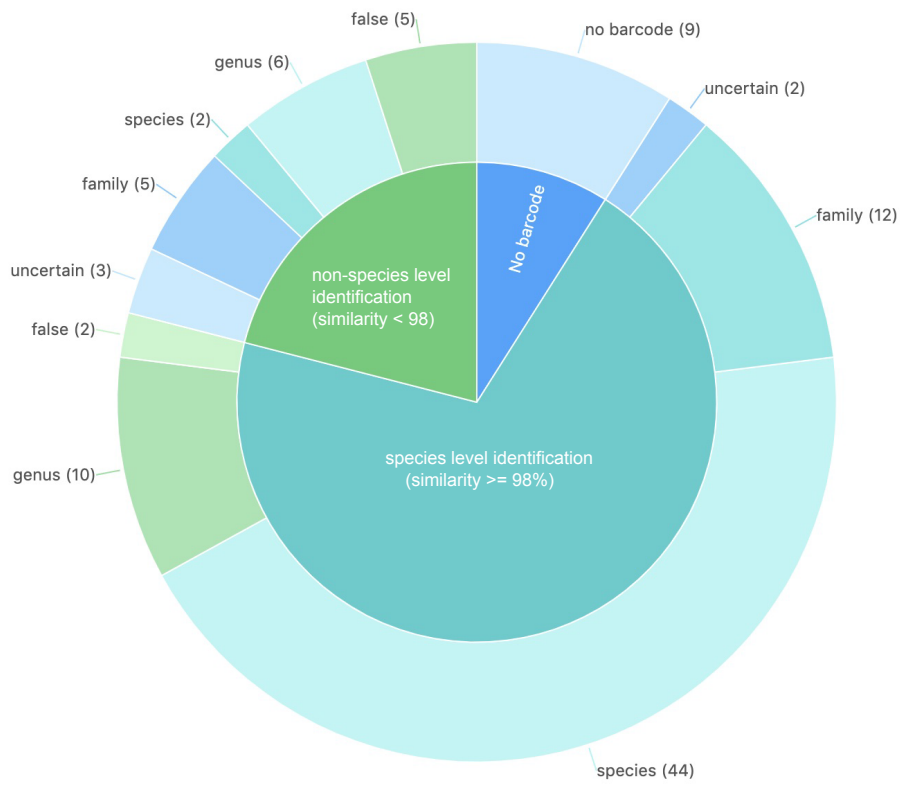

Supplement: Supplementary file 1 — Additional file 1: Figure S1. Read counts of the Sanger barcode failed samples. Stars indicate samples of which short amplicon(s) was detected in the HIFI assemblies. Short amplicons are those clusters of abundance > 10 and of length < 600 bp. The bar plot demonstrates the number of assigned reads for the barcode failed samples. The red dashed line shows the average value of all the successful samples and no significant difference was detected between the two groups (P value of 0.232, Student’s t-Test). Figure S2. Discrepancies between Sanger sequences and HIFI-SE barcodes. Entropy weight was calculated based on the strength of read depth by aligning the SE400 reads onto the assembled HIFI-SE barcodes, showing differences between ambiguous Sanger base-calling and specific nucleotide identified in HIFI-SE barcodes (A) and potential heteroplasmy (B). In addition, several N bases were present of insertion in Sanger sequence (C), also two N bases in HIFI sequences (D). Figure S3. Comparison of molecular and morphological identification. [file 12864_2020_7255_MOESM1_ESM.pdf]
