# Supplementary material for: Efficient COI barcoding using high throughput single-end 400 bp sequencing
Source: BMC Genomics. 2020 Dec 4;21:862. doi: 10.1186/s12864-020-07255-w (PMC7716423; doi:10.1186/s12864-020-07255-w)
Supplement: Supplementary file 5 — Additional file 5. A note for taxonomy identification issue of sample #035 in plate #4. [file 12864_2020_7255_MOESM5_ESM.pdf]

The phylogeny tree revealed that some specimens could be wrongly identified based on an inadequate database in specific linage. For example, the best hit of #035 in Plate #4 with 100% similarity in BOLD database belongs to Crambidae family, however, the second hit with 99.85% similarity belongs to Erebidae family. This kind of wrong placement are prone to be happened among early-release records, which suggests a new record of specimen need to be carefully reviewed when add to a database, also indicating that morphological identification is still important.

Access in Apr. 2019

Display: Top 20

Top 20 Matches

Phylum	Class	Order	Family	Genus	Species	Subspecies	Similarity (%)	Status
Arthropoda	Insecta	Lepidoptera	Crambidae	*Cirrhochrista*	*brizoalis TW*		100	Early-Release
Arthropoda	Insecta	Lepidoptera	Erebidae	*Spirama*	*retorta*		99.85	Published
Arthropoda	Insecta	Lepidoptera	Erebidae	*Spirama*	*retorta*		99.85	Published
Arthropoda	Insecta	Lepidoptera	Erebidae	*Spirama*	*retorta*		99.85	Published
Arthropoda	Insecta	Lepidoptera	Erebidae	*Spirama*	*retorta*		99.85	Published
Arthropoda	Insecta	Lepidoptera	Erebidae	*Spirama*	*retorta*		99.85	Published
Arthropoda	Insecta	Lepidoptera	Erebidae	*Spirama*	*retorta*		99.85	Published
Arthropoda	Insecta	Lepidoptera	Erebidae	*Spirama*	*retorta*		99.69	Published
Arthropoda	Insecta	Lepidoptera	Erebidae	*Erebus*	*sp. 4*		99.69	Early-Release
Arthropoda	Insecta	Lepidoptera	Erebidae	*Spirama*	*retorta*		99.69	Early-Release
Arthropoda	Insecta	Lepidoptera	Erebidae	*Spirama*	*helicina*		99.67	Published
Arthropoda	Insecta	Lepidoptera	Erebidae	*Hypopyra*	*vespertilio*		99.66	Private
Arthropoda	Insecta	Lepidoptera	Erebidae	*Spirama*	*retorta*		99.62	Published
